# The Role of Guilt Feelings in the Development of the Burnout Process: The Influence on Psychosomatic Problems

**DOI:** 10.3390/bs14121196

**Published:** 2024-12-13

**Authors:** Pedro Gil-LaOrden, Mary Sandra Carlotto, Pedro R. Gil-Monte

**Affiliations:** 1 Unidad de Investigación Psicosocial de la Conducta Organizacional (UNIPSICO), Faculty of Psychology, Universitat de València, Av. Blasco Ibáñez 21, 46010 Valencia, Spain; pedro.gil-monte@uv.es; 2 Postgraduate Program in Social, Work and Organizational Psychology, Universidade de Brasília, Campus Universitário Darci Ribeiro, ICC Sul, Brasília 70900-910, Brazil; mary.carlotto@unb.br

**Keywords:** burnout, feelings of guilt, psychosomatic problems, Spanish Burnout Inventory, occupational stress, teachers

## Abstract

Burnout is a psychological consequence of prolonged work-related stress. Previous studies have concluded that guilt feelings could explain the development of the burnout process and its relationship with other health disorders. The aim of this study was to evaluate the mediating role of guilt feelings in the relationship between burnout and psychosomatic problems. The sample comprised 714 Brazilian teachers (82.10% women). Burnout was assessed using the Spanish Burnout Inventory (SBI). The hypotheses were evaluated together using a path model to test the mediating role of guilt feelings in the development of burnout and its relationship with psychosomatic problems. Two models were constructed: the hypothesized model (i.e., indolence → guilt → psychosomatic problems) vs. the alternative model (i.e., indolence → psychosomatic problems → guilt). According to the results, the hypothesized model obtained a satisfactory fit to the data, whereas the alternative model’s fit was found to be inadequate. We concluded that the hypothesized model was a good representation of the relationship among burnout, guilt feelings and psychosomatic problems. We recommend taking into consideration feelings of guilt to improve the diagnosis of burnout.

## 1. Introduction

According to the World Health Organization [1], burnout is a consequence of chronic work-related stress that has not been successfully managed. It has been included in the International Classification of Diseases and Related Health Problems (ICD-11), and classified as a factor influencing health status or contact with health services (QD85). It is considered as a circumstance rather than a disease and it is not considered a medical condition. It refers specifically to phenomena in the labor context and should not be applied to experiences in other life domains. Therefore, burnout should be considered a health disorder, but not a psychiatric syndrome or a mental illness. It is characterized by three dimensions: (1) feelings of energy depletion; (2) feelings of negativism related to one’s job; and (3) a sense of ineffectiveness and low accomplishment (ICD-11). This definition currently finds considerable consensus in the field and is most frequently used to evaluate burnout. It takes as its reference the psychometric model of the Maslach Burnout Inventory (MBI) [2].

In addition, several studies have suggested the existence of profiles and alternative theoretical frameworks for burnout [3,4,5,6,7], and latent profiles for burnout dimensions [8,9,10,11,12,13]. Studies on latent profiles are not based on a theoretical model, but rather on a psychometric model. Most of these studies used the theoretical definition provided by the WHO [1] and the MBI to differentiate burnout profiles [11,12,14]. For example, Leiter and Maslach [11], working with the MBI, identified five burnout profiles, labeled as burnout, disengaged, overextended, ineffective, and engagement. However, other studies have obtained four profiles [15,16]. Some studies have used alternative profiles to those of the MBI for assessing burnout, but these lacked a theoretical model that explains the syndrome’s development process [10,13,17,18].

### 1.1. Theoretical Background

Gil-Monte [19] defined burnout as a syndrome resulting from chronic occupational stress of an interpersonal nature, which appears in professionals in service organizations. It is characterized by loss of enthusiasm towards the job, psychological exhaustion, indolence, and, in some cases, feelings of guilt may appear and lead to more serious health consequences. Three of them are partially similar to the definition of burnout given by the World Health Organization in the ICD-11: psychological exhaustion is similar to emotional exhaustion, indolence overlaps with depersonalization and cynicism, and low enthusiasm towards the job partially overlaps with lack of accomplishment. Feelings of guilt is a new dimension to burnout syndrome [20]. These symptoms constitute the four dimensions of the Spanish Burnout Inventory (SBI) [19].

According to Gil-Monte [19], burnout progresses in parallel from low enthusiasm towards the job and psychological exhaustion to indolence. Indolence is viewed as a dysfunctional rather than an effective coping strategy, which is employed after the reappraisal stage. This approach considered the model of attitudes developed by Eagly and Chaiken [21], and it took into consideration the mediating role of emotional and cognitive experiences in the relationship between perceived job stress and attitudinal outcomes.

Guilt is a social emotion characterized by an unpleasant feeling arising when we perceive that our actions have violated a moral or ethical standard and involves a negative self-assessment of our behavior [22]. It is a prosocial emotion, which motivates behavior adapted to social and cultural rules. It has a potentially positive function within social interactions by stimulating pro-social behaviors and promoting positive actions towards those who have been wronged to amend any damage caused [23].

Guilt has been incorporated by the model of Gil-Monte to explain two profiles of burnout. In both profiles, indolence is considered as a coping strategy used to alleviate cognitive (i.e., lower enthusiasm towards the job) and emotional (i.e., psychological exhaustion) deterioration. But this coping strategy yields different outcomes for different professionals. While some professionals effectively manage strain levels using the attitudes and behaviors of indolence (Profile 1), others find it uncomfortable, leading to increased guilt feelings and more severe burnout (Profile 2).

Profile 1 characterizes professionals who do not develop high levels of guilt. They experience moderate job-related stress, resulting in noticeable discomfort that, while not debilitating, does impact work performance to some degree. This profile is marked by low enthusiasm towards the job, coupled with high levels of psychological exhaustion and indolence. On the contrary, professionals who develop Profile 2 feel more intense burnout symptoms and health disorders [24]. They perceive the use of indolence as a non-adaptative coping strategy. When these professionals see themselves as becoming dehumanized, they experience high levels of guilt and reaffirm their commitment to other people (e.g., students and their parents) and professional involvement as a way of mitigating their feelings of guilt, mending past transgressions and any damage caused [25,26,27]. However, negative working conditions do not change (e.g., negative workplace relationships), their results do not improve, and job stress levels do not decrease, giving rise to more indolence (a negative coping strategy) and more intense feelings of chronic guilt [28,29,30]. Profile 2 suggests a mediator role for feelings of guilt in the relationship between burnout and more severe health disorders such as depression [4] because they work as a “trigger variable” in the progress of burnout. These profiles have been validated through cluster analysis [9] and predictive validity studies [24].

Self-efficacy has been identified as a relevant variable for professional development and well-being in the teaching profession, and for explaining the increasing teacher shortages observed in recent years [31]. Perceived self-efficacy refers to “beliefs in one’s capabilities to organize and execute the courses of action required to produce given attainments” [32]. An efficacy expectation refers to an individual’s belief in their ability to perform the necessary actions to achieve the desired results. Teacher self-efficacy refers to the teacher’s belief in their capabilities to effectively cope with the responsibilities, duties, and obstacles associated with the professional role, including didactical tasks and addressing disciplinary problems in the classroom [33,34].

Self-efficacy has been identified as a relevant resource in preventing burnout [35,36,37] in educational settings. The relationship among these variables was found to be negative [38,39]. The results of previous studies have indicated that self-efficacy is primarily positively associated with personal accomplishment, and the relationship is weaker and negatively associated with the emotional component of burnout [40,41,42,43,44].

On the other hand, social support has been identified as a relevant psychosocial resource at work to prevent burnout [36,45,46,47]. Previous studies in the literature have shown a positive relationship between social support and the cognitive dimension of burnout –i.e., personal accomplishment or enthusiasm towards the job– and a negative relationship with the emotional dimension—i.e., psychological exhaustion [48,49,50,51].

Previous studies have identified health disorders that are positively associated with burnout among teachers. Chronic burnout is a serious health disorder, leading to mental health problems [52], anxiety [53,54], sleep disturbances [55,56], cardiovascular disease, musculoskeletal disorders, respiratory problems, gastroenteritis, and migraines [57].

### 1.2. Teachers’ Burnout

Studies in education have concluded that burnout in non-university teachers is a serious problem around the world [48,58,59,60]. Teaching has become a high-risk profession for this syndrome due to the challenging working conditions in education. The demanding nature of the job, which involves constant interaction with people, exposes teachers to an increased risk of developing burnout. Teaching involves dealing with disruptive or difficult students, satisfying high expectations from parents and administrators, coping with excessive workloads, having high levels of commitment to the job, and facing the fact that most educational centers do not take into consideration the work–life balance of their teachers [61]. Moreover, principals focus on external inspections, accountability, and a blame culture, which increases job stress and burnout [62].

According to estimations by Marsollier et al. [59], the prevalence of burnout in Latin American teachers was higher than 30%, for high and very high (critical) levels. Carlotto and Câmara [48] found that the prevalence of critical burnout among Brazilian teachers was 25.80%, which indicated a high prevalence of burnout in Latin American and Brazilian teachers.

### 1.3. The Present Study

**Figure 1:**
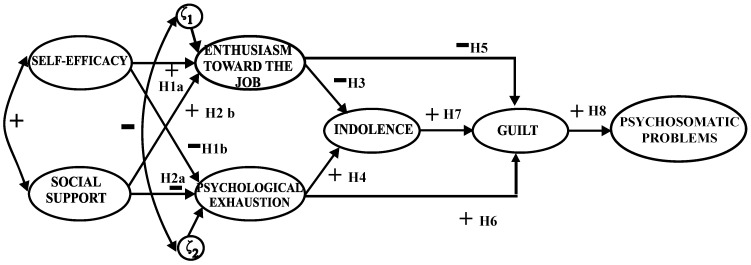
Hypothesized model.

Based on the previous results, the aim of this study was to evaluate the mediator role of guilt feelings in the process of burnout and the relationship between burnout and psychosomatic problems, and to test the cross-national validation of the model of burnout developed by Gil-Monte [19]. The hypotheses were evaluated together using a path model to test the mediator role of guilt (as a symptom of burnout) in the process of burnout, and its relationship with levels of psychosomatic problems (Figure 1). We expected that indolence would be positively related to guilt (Hypothesis 7), and that guilt would be positively related to psychosomatic problems (Hypothesis 8).

Similarly, the possible reversed effect was also investigated, that is, whether psychosomatic problems mediate the relationship between indolence and guilt (alternative model). Psychosomatic problems could increase the difficulty of development in a demanding role, which ultimately increases the feelings of guilt. People with chronic psychosomatic problems may experience feelings of guilt, stemming from the physical and psychosocial symptoms of their illness. These feelings can arise from various factors, including the inability to perform daily activities independently and the impact on their work relationships [63]. Teachers can feel guilt related to a subjectively perceived responsibility, for inaction and not achieving their goals, and for not fulfilling a responsibility because of their own diseases or traumatic experiences [25].

Two path models were utilized to analyze the relationships among the variables. The order of the guilt–psychosomatic problems relationship established the distinction between the models. According to the hypothesized model, the feelings of guilt will occur prior to the psychosomatic problems (i.e., indolence → guilt → psychosomatic problems), while the alternative model states that the psychosomatic problems will occur prior to the feelings of guilt (i.e., indolence → psychosomatic problems → guilt).

## 2. Materials and Methods

### 2.1. Participants

The sample included 714 non-university teachers from the south of Brazil (men, 115; women, 586) (82.10% women). The mean age was 39.32 years (SD = 10.01, age range = 19–67 years). Regarding the type of contract, 66.80% of the sample (n = 477) were tenured teachers, and 30.10% (n = 215) were temporary teachers. The mean years of seniority in the profession of all the participants was 13.80 years (SD = 9.30, seniority range = 1–48 years), working in public institutions (63.50%) and working exclusively in teaching (62.1%).

### 2.2. Instruments

*Sociodemographic data*. All respondents were given a standard set of sociodemographic variables to obtain information about gender, age, contract type, and professional seniority.

*Self-efficacy* was measured using an adaptation of the *General Self-efficacy Scale* [64]. The adapted scale consisted of 8 items, similar to those of the original version but referring to work environment (e.g., When I am confronted with a problem in my job, I can usually find several solutions) (α = 0.87). The scale is a self-assessment tool designed to evaluate optimistic self-beliefs in coping with challenging demands at work.

*Social support* was evaluated using the scale from the *UNIPSICO questionnaire* [65]. This scale consists of 6 items that measure the emotional and the instrumental support from colleagues, direct supervisor, and principal (e.g., How often do your co-workers help you when problems arise at work? Do you feel appreciated by your direct supervisor at work?) (α = 0.85).

*Burnout* was measured using the *Spanish Burnout Inventory (SBI)* [19]. This questionnaire is a 20-item scale, divided into four subscales: *Enthusiasm towards the job*, which refers to an individual’s desire to reach work-related goals because it is a source of personal accomplishment (5 items, e.g., I think my job gives me positive experiences) (α = 0.83); *Psychological exhaustion*, which is the appearance of emotional and physical exhaustion, resulting from the demands of interacting with people experiencing problems (4 items, e.g., I feel I am overwhelmed by work) (α = 0.80); *Indolence*, which refers to the appearance of negative attitudes and behaviors of indifference and cynicism towards the organization’s clients (6 items, e.g., I treat some people in my work with indifference) (α = 0.80); and, *Guilt*, which manifests as feelings of remorse about negative workplace attitudes and behaviors, particularly towards the people with whom the professionals establish work relationships (5 items, e.g., I feel bad about some of the things I have said at work) (α = 0.81). Low levels of enthusiasm towards the job, with high levels of psychological exhaustion and indolence, as well as guilt, indicate high levels of burnout. The version adapted for the Brazilian Portuguese language was applied [66,67].

*Psychosomatic problems* were evaluated by the psychosomatic disorders scale of the *UNIPSICO questionnaire* [24]. Items on this scale include different work-related psychosomatic problems (9 items, e.g., headaches, sleep quality, and anxiety, e.g., Do you have a headache?) (α = 0.90).

All scales were translated into Brazilian Portuguese using the back translation procedure [66]. Participants answered the items on all the scales on a 5-point frequency scale ranging from “Never” (0) to “Very frequently: Every day” (4).

### 2.3. Data Collection

The study respected the fundamental principles of the Declaration of Helsinki [68] and was approved by the Institutional Review Board of Pontifical Catholic University of Rio Grande do Sul, with particular emphasis on the confidentiality and non-discrimination of participants. Resolution 196 of the National Health Council (NHC) guided the ethical procedures. The participation in the study was voluntary and anonymous. Prior to the start of the assessment, the school principal was contacted to obtain the authorization to carry out the study and design the data collection. Teachers were informed about the purpose of the study, and it was clarified that their participation would not result in any individual or organizational assessment consequences. The questionnaire was distributed to teachers at the beginning of their work day, and they were asked to submit the completed questionnaire by placing it in a box in the teachers’ room at the end of the day. Teachers expressed their agreement in a free and informed consent form before completing the questionnaire.

### 2.4. Data Analysis

To calculate the descriptive statistics, reliability and Pearson correlations the SPSS program (version 28.0) were used. To test the models, a structural equation analysis was carried out using the AMOS 25.0 program. The maximum likelihood estimation method was applied to test the models. Because the χ^2^ test is sensitive to sample size, alternative fir indices were proposed. The *Goodness of Fit Index* (GFI) is a measure of the relative degree of variance and covariance accounted by the model. The *Adjusted Goodness of Fit Index (AGFI)* is a correction of the GFI based upon degrees of freedom. The *Non-Normed Fit Index* (NNFI) and the *Comparative Fit Index* (CFI) compared the relative fit of the hypothesized model with that of a baseline, or null, model. They should be greater than 0.90 [69]. However, some studies [70] have recommended a cutoff value of 0.95 as rule of thumb for GFI, AGFI [71] (p. 107), NNFI [72], and CFI [72,73]. The *Root Mean Square Error of Approximation* (RMSEA) quantified the error of the approximate fit, and the *Standardized Root Mean Squared Residual* (SRMR) was the square-root of the difference between the sample covariance matrix residuals and the proposed model. For both indices, values less than 0.05 indicated a close model fit, and values less than 0.08 suggested a reasonable model-data fit [74,75,76]. Values greater than 0.10 indicated poor fit [75]. For testing mediation and indirect effects, bootstrapping was carried out, with the number of bootstrap samples set at 5000 and 95% confidence intervals [77].

## 3. Results

Descriptive statistical analyses and internal consistency values are shown in Table 1. All scales showed good reliability values, with Cronbach’s higher than 0.70. Regarding the skewness and kurtosis values, the variables fitted the normal distribution because all values ranged between +1 and −1.

Table 2 shows the Pearson correlations among the variables included in this study. All correlations followed the hypothesized direction, and they were significant at *p* < 0.01 or lower, except for social support and guilt correlation, which was not significant. For self-efficacy and social support, higher levels were related to lower burnout, and higher levels of burnout were related to higher levels of psychosomatic problems.

The hypothesized model resulted in a significant Chi^2^ value (χ^2^_(9)_ = 168.645, *p* < 0.001), suggesting an inadequate model fit. While the GFI indicated a good fit with the data (0.942), the fit was not acceptable according to the values achieved by the other indices (Table 3). The modification indices were reviewed to identify where linear constraints might be released to improve the model fit. The highest value suggested freeing the path associated with the variables: psychological exhaustion → psychosomatic problems (MI = 116.29). Freeing this path followed a theoretical basis, as the literature concluded that there is a significant and positive relationship between psychological exhaustion and psychosomatic problems [78,79]. Additionally, it did not invalidate this study’s objective, as it continued to test the mediating role of feelings of guilt in the burnout process.

After this, the revised model—i.e., the “hypothesized model” revised—was estimated again, the final fit indices were as follows: χ^2^_(8)_ = 14.647, *p* > 0.05); GFI = 0.994; AGFI = 0.980; NNFI = 0.985; CFI = 0.994; RMSEA = 0.034 (90% CI = 0.000–0.061); and SRMR = 0.023 (Table 3). These values indicated a very good fit of the data to the overall revised hypothesized model.

**Figure 2:**
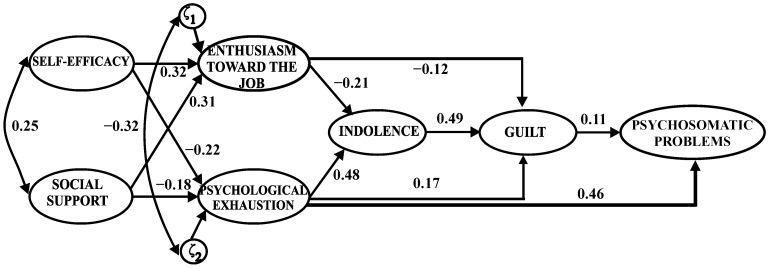
Standardized coefficients for the hypothesized model revised. Note. All relationships were significant at *p* ≤ 0.001.

All the paths were significant at *p* ≤ 0.001 (Figure 2), and all hypotheses were confirmed. Specifically, self-efficacy was positively related to enthusiasm towards the job (*γ* = 0.32) and negatively related to psychological exhaustion (*γ* = −0.22). Social support presented a positive relationship with enthusiasm towards the job (*γ* = 0.31) and a negative relationship with psychological exhaustion (*γ* = −0.18). Regarding the hypotheses related to the burnout process, enthusiasm towards the job showed a negative relationship with indolence (*β* = −0.21), and psychological exhaustion showed a positive relationship with indolence (*β* = 0.48). In addition, enthusiasm towards the job presented a negative relationship with guilt (*β* = −0.12); psychological exhaustion obtained a positive relationship with guilt (*β* = 0.17); and a significant positive relationship was found between indolence and guilt (H7) (*β* = 0.49). Lastly, a significant positive relationship was found between guilt and psychosomatic problems (H8) (*β* = 0.11), and psychological exhaustion showed a positive relationship with psychosomatic problems (*β* = 0.46) (Figure 2). The revised model explained 32% of the variance in guilt and 27% of the variance in psychosomatic problems.

Using bootstrapping, the standardized indirect effect of indolence on psychosomatic problems was 0.09 (*p* < 0.001; bias corrected 95%, CI: 0.042 to 0.141), indicating that the relationship between indolence and psychosomatic problems was mediated by the variable of guilt (H7 and H8 were confirmed). However, the direct effect of indolence on psychosomatic problems remained significant (*β* = 0.25, *p* < 0.001; bias corrected 95%, CI: 0.161 to 0.332), indicating the partial mediation effect of the guilt variable.

The alternative model yielded a significant Chi2 value (χ^2^_(9)_ = 223.829, *p* < 0.001), which indicated an insufficient model fit according to this index. The fit to the data was adequate according to the GFI (0.926), but it was not acceptable according to the values achieved by other indices (Table 3). The modification indices were evaluated to improve the model fit. It appeared that releasing the constrained paths linking indolence with guilt (MI = 150.028, par change = 0.454), and psychological exhaustion with guilt (MI = 48.991, par change = 0.216) would improve the model fit. In addition, a review of the parameter estimations indicated that the relation between enthusiasm towards the job and psychosomatic problems was not significant (*β* = 0.03, *p* = 0.390).

With these results, we considered whether changing the model according to the modification indices would be a valid theoretical approach, because the highest modification index value suggested the freeing of the path associated with the variables indolence → guilt; however, there was no reason for considering this to be an adequate theoretical redesign for the alternative model.

**Figure 3:**
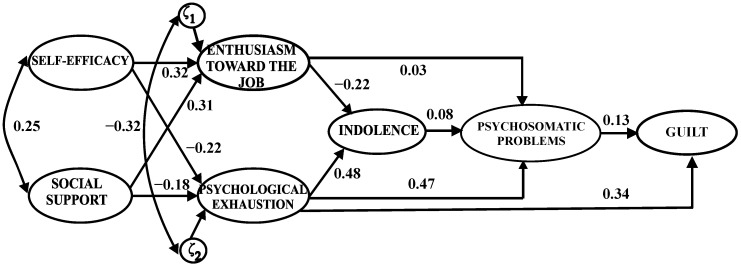
Standardized coefficients for the alternative model revised.

In light of these results, an alternative revised model was evaluated, removing the non-significant relationship and adding the path psychological exhaustion → guilt (Figure 3). The fit indices were as follows: χ^2^_(8)_ = 154.856, *p* < 0.001; GFI = 0.945; AGFI = 0.806; NNFI = 0.678; CFI = 0.877; and RMSEA = 0.160 (90% CI = 0.139–0.183) (Table 3). According to these results, the fit to the data of the alternative model redesign was not adequate.

## 4. Discussion

This research sought to evaluate how guilt feelings mediate the burnout process and its relationship to psychosomatic problems, and to test the cross-national validation of the model of burnout developed by Gil-Monte [19]. The results supported that the relations represented in the hypothesized model, i.e., indolence → guilt → psychosomatic problems, are a good depiction of the burnout process and its relationship with psychosomatic problems. However, the relations depicted in the alternative model, i.e., indolence → psychosomatic problems → guilt, are not an adequate representation of the burnout process. The results provided support for the mediator role of feelings of guilt in the development of burnout and its consequences on the health of Brazilian teachers. This pattern suggests that higher levels of indolence lead to heightened feelings of guilt, which are, in turn, associated with the development of higher levels of psychosomatic problems, in line with the development of Profile 2 of burnout, hypothesized by the model of Gil-Monte.

Previous studies reached similar conclusions based on samples of teachers from Portugal and Spain [78]. In addition, the model has been supported for symptoms of depression in employees working with intellectually disabled people [4], and for alcohol intake and tobacco use as a consequence of troubles related to work in a sample of Chilean administration and customer service workers [80]. However, those previous studies did not test an alternative model, which is what makes this study original. It has also added new knowledge to the development of the burnout process, according to the model designed by Gil-Monte, by highlighting the relevance of developing feelings of guilt in explaining why job burnout leads to more serious psychosomatic problems.

The SBI theoretical model proposed two burnout development profiles. Both profiles feature indolence as a coping strategy that is employed in response to diminished enthusiasm towards the job and high psychological exhaustion. The feelings of guilt work as a “trigger variable” in the progress of burnout. Therefore, low or medium levels of guilt can be managed by personal coping strategies, i.e., Profile 1. However, when the feelings of guilt reach very high or critical levels, which cannot be managed by the individual without professional help, they accelerate the development of burnout into more serious health disorders, such as depression or psychosomatic problems, i.e., Profile 2 [24].

In addition, the results followed along the lines of other studies, which have pointed to self-efficacy [35,46] and social support [45,46] being relevant variables in preventing burnout in non-university teachers. When teachers show high self-efficacy and satisfaction with the perceived social support that they receive at work, their risk of burnout decreases.

It is necessary to highlight some limitations of this study. First, the cross-sectional data analysis presented limitations in determining the causal relationship between guilt and psychosomatic problems, making it challenging to establish whether guilt leads to psychosomatic problems or vice versa. Longitudinal studies are needed to draw conclusions on this issue. Second, the data derived from questionnaires increased the likelihood of common method variance effects. We employed various strategies to reduce the influence of these biases [81]: (a) participants’ confidentiality was maintained; (b) participants did not know which items were associated with which scales; (c) back translation was carried out to minimize item ambiguity; and (d) an analysis using Harman’s single factor test revealed that the total variance explained by one factor (32.81%) was less than 50% of the recommended threshold, indicating that common method bias is unlikely to significantly impact the study’s findings. Third, participant selection was non-random.

## 5. Conclusions, Practical Implications, and Future Recommendations

The results of this study support the theoretical studies that have concluded that guilt is a significant variable in understanding the development of burnout [82,83,84,85]. This indicates that the feelings of guilt experienced by teachers play a significant role in explaining the different profiles of burnout progression [9] and its relationship with psychosomatic problems, which are closely associated with the development of guilt over time.

Feelings of guilt should be considered a symptom of burnout in order to achieve a more comprehensive diagnosis, distinguishing between individuals affected by the syndrome and recognizing its impact on health-related issues. While our research focused on a sample of teachers, burnout is also common among professionals in other helping professions (e.g., health, social services, and public servants). It may provide information for therapists to aid in the prevention, diagnosis, and treatment of different types of burnout syndrome—i.e., Profile 1 vs. Profile 2.

This study could also be useful in detecting the need for intervention programs to improve the social environment at educational centres and the need to train teachers in techniques for dealing with stress and burnout [86]. Principals and school administrators might help to promote collaborative relationships among teachers through programs for increasing social support, and by enhancing teachers’ skills and capacities related to self-efficacy to prevent burnout and psychosomatic problems.

Future research should continue to investigate which situational (e.g., socialization in the professional role) [87,88] and individual factors (e.g., prosocial preferences and moral values) [89] cause guilt in the process of burnout and burnout Profile 2.

## Figures and Tables

**Table 1:** Descriptive statistics for scales in this study and Cronbach’s alpha values.

	M	SD	Min	Max	Skewness	Kurtosis	Alpha
1. Self-efficacy	2.92	0.59	1.00	4.00	−0.38	0.22	0.87
2. Social support	2.48	0.85	0.00	4.00	−0.13	−0.61	0.85
3. Enthusiasm towards job	3.01	0.80	0.20	4.00	−0.69	−0.21	0.83
4. Psychological exhaustion	1.63	0.89	0.00	4.00	0.43	−0.17	0.80
5. Indolence	1.01	0.74	0.00	4.00	0.95	0.78	0.80
6. Guilt	1.11	0.77	0.00	4.00	0.66	0.30	0.81
7. Psychosomatic problems	1.38	0.93	0.00	4.00	0.60	−0.23	0.90

**Table 2:** Pearson correlations among the study variables.

	1	2	3	4	5	6	7
1. Self-efficacy	1						
2. Social support	0.25	1					
3. Enthusiasm towards job	0.40	0.39	1				
4. Psychological exhaustion	−0.26	−0.24	−0.42	1			
5. Indolence	−0.26	−0.14	−0.41	0.57	1		
6. Guilt	−0.13	−0.00	−0.15	0.40	0.54	1	
7. Psychosomatic problems	−0.15	−0.12	−0.20	0.51	0.34	0.30	1

Note. Values > |0.12| were significant at *p* < 0.001, and values ≤ |0.12| were significant at *p* < 0.01, except social support and guilt correlation, which was not significant.

**Table 3:** Fit indices for the study models.

	χ^2^	df	RMSEA_(90% CI)_	SRMR	GFI	AGFI	NNFI	CFI
Hypothesized model	168.645 *	9	0.158_(0.137–0.179)_	0.093	0.942	0.820	0.688	0.866
Hypothesized rev. model	14.647	8	0.034_(0.000–0.061)_	0.023	0.994	0.980	0.985	0.994
Alternative model	223.829 *	9	0.183_(0.163–0.204)_	0.100	0.926	0.770	0.581	0.820
Alternative rev. model	154.856 *	8	0.160_(0.139–0.183)_	0.062	0.945	0.806	0.678	0.877

* *p*< 0.001. Note 1. χ^2^—Chi-square; df—degrees of freedom; RMSEA_(CI)_—root mean square error of approximation (90% confidence intervals); SRMR—standardized root mean squared residual; GFI—goodness-of-fit index; AGFI—adjusted goodness of fit index; NNFI—non-normed fit index; and CFI—comparative fit index.

## Data Availability

The raw data supporting the conclusions of this article will be made available by the authors upon request.
